# Space Human–Robot Interaction with Gaze Tracking Based on Attention Mechanism

**DOI:** 10.3390/biomimetics11020103

**Published:** 2026-02-02

**Authors:** Lihong Dai, Jinguo Liu, Zhaojie Ju

**Affiliations:** 1 School of Electrical and Electronics Engineering, Hubei Polytechnic University, Huangshi 435003, China; dailihong@hbpu.edu.cn; 2 State Key Laboratory of Robotics, Shenyang Institute of Automation, Chinese Academy of Sciences, Shenyang 110016, China; liujinguo@sia.cn; 3 Institutes for Robotics and Intelligent Manufacturing, Chinese Academy of Sciences, Shenyang 110169, China; 4 Key Laboratory for Biomedical Engineering of Ministry of Education, College of Biomedical Engineering and Instrument Science, Zhejiang University, Hangzhou 310007, China

**Keywords:** gaze tracking, space human–robot interaction, attention mechanism, radio frequency communication

## Abstract

Gaze is a natural and rapid non-verbal interaction mode, particularly well-suited for human–robot interaction in busy space environments. However, the space human–robot interaction based on gaze is still in its infancy. Therefore, this paper conducts a preliminary exploration in this area. Using the AAR-2, a free-flying astronaut-assistant robot, as the platform, we establish a gaze tracking database, construct a gaze tracking model based on an attention mechanism, and develop a human–robot interface. When the astronaut gazes at a control button on the interface, the corresponding control instruction is transmitted to the STM32 controller within the AAR-2 via radio frequency communication. Subsequently, the AAR-2 is propelled by ducted fans to perform the corresponding action. At the same time, the AAR-2 feeds back its operational state to the astronaut, thereby enabling space human–robot interaction. In the system, we achieve an effective gaze tracking model with high accuracy and implement an efficient image preprocessing method with high real-time performance. The experimental results demonstrate that the system can meet the actual requirements for accuracy and real-time processing.

## 1. Introduction

In human–robot interaction (HRI), diverse interaction modalities exist, including tactile feedback, vocal commands, gesture recognition, facial expression analysis, and the brain–robot interface (BRI). However, in space environments where astronauts’ operational time is critically constrained, efficiency becomes the major consideration. Non-verbal interaction modes, particularly gaze tracking, offer significant advantages by enabling rapid, intuitive control without manual operation. Despite its potential, gaze-based interaction in space remains underexplored. Therefore, we have conducted in-depth research on gaze-based space HRI. On the one hand, to enhance the accuracy of gaze tracking, we integrated an attention mechanism into the network model and proposed a gaze tracking method named BinocularFullAttention. On the other hand, to validate real-time performance, we leveraged the astronaut-assistant robot AAR-2 to carry out space HRI simulation experiments, achieving real-time control of AAR-2’s movements through astronaut gaze. Related work on HRI and gaze tracking is introduced as follows.

### 1.1. Human–Robot Interaction Related Work

#### 1.1.1. Human–Robot Interaction Mode

In the process of HRI, robots often function in accordance with the control instructions sent by users, cooperate with people to complete the designated tasks, or interact and communicate with people. Moreover, for different application scenarios, there are various interaction modes between human and robot.

For example, in tactile HRI, challenges like limited sensitivity, durability problems, and slow response emerge, ultimately leading to an suboptimal interaction experience [1]. Voice is a natural way of interaction [2]. However, in a complex interactive environment, voice is often unreliable due to the influence of noise. Gesture is another natural way of interaction [3], and especially hand gesture is now favored by many researchers. Data gloves or cameras are utilized to collect signals or images to recognize hand gestures, in order to implement HRI [4,5,6,7]. Moreover, the combined control of gesture and voice is used to achieve natural interaction in [8]. However, hand gesture is often inconvenient for astronauts to use for interaction due to their busy work. In addition, new ways of HRI have emerged, such as expression and brain–robot interface. In [9,10], different expressions are recognized and applied to social robots. Human expressions are very rich, especially micro-expression recognition is very difficult, so it is very challenging to carry out HRI by facial expression recognition [11]. For HRI using BRI, users stick electrodes on their heads or use head-mounted devices with a brain–computer interface to control robots by motion imagination [12,13]. However, the application of BRI requires a high degree of attention, which results in cognitive burden and mental fatigue.

The gaze can be interpreted as the direct output of the human brain, and compared with detecting the human brain by Electroencephalogram (EEG), it is easier, faster, and more accurate to detect the movement of human eyes. As a result, some gaze-based interactions appear. In [14], the direction acquired by gaze tracking and the speed obtained by the remote controller collectively reflect the operator’s intentions, which are applied in aerial teleoperation for skilled and unskilled operators. In [15], by virtue of a 360° camera and an eye-tracking device, gaze is used to control an electric wheelchair. In [16], a deep neural network model based on gaze tracking is proposed to recognize the user’s intention and predict the desired actions to control the robot to perform daily tasks. In [17], by capturing the operator’s eye movements, the robot ultrasound system is guided to track the correct blood vessel at the vascular bifurcation, effectively reducing the difficulty of traditional medical ultrasound examinations. However, in the above methods, eye trackers are used. These devices are not only intrusive to people and not convenient for HRI, but also the cost is high. Therefore, in the process of HRI, there are also some gaze tracking methods that do not use head-mounted devices. In [18], the gaze is used to control the needle in the robot navigation system, and five gaze directions (up, down, left, right, and center) are used, where the left and right ones control the movement direction of the needle, and the up and down ones control its insertion and extraction. In [19], the robot predicts and tracks the subject’s gaze with 15 positions and 8 directions by the probability-based method, which is applied for the teleoperation of the disabled or the elderly in the home environment. However, in the above two methods, discrete gaze directions are adopted, which will inevitably lower the accuracy of gaze tracking. In this paper, we adopt remote gaze tracking based on continuous gaze point positions, eliminating the limitations of head-mounted devices. By mimicking the natural fidelity of human eye movements, this remote method offers a biomimetic alternative to constrained head-mounted systems. Unlike discrete gaze direction approaches, our continuous tracking captures subtle eye movements, thereby enhancing gaze tracking accuracy. In [20], the robot recognizes the human head and body movement as well as the gaze in order to better carry out HRI, in which the head pose is used to approximate the gaze. In [21], it is discussed that gaze tracking is better than head tracking in human–robot cooperation, which also shows the advantages of gaze tracking.

To sum up, as a natural and rapid means of interaction, gaze has its distinct advantages. Gaze can directly reflect people’s thoughts and intentions, and is a non-verbal interaction mode, which is not affected by the interference such as noise. It can also free up people’s hands and is more suitable for complex space HRI scenarios. Although gaze is gradually being used in various HRI, its application in space is still in its infancy. This paper makes a preliminary exploration on the application of gaze tracking in space HRI.

#### 1.1.2. Space Human–Robot Interaction Platform

**Figure 1:**
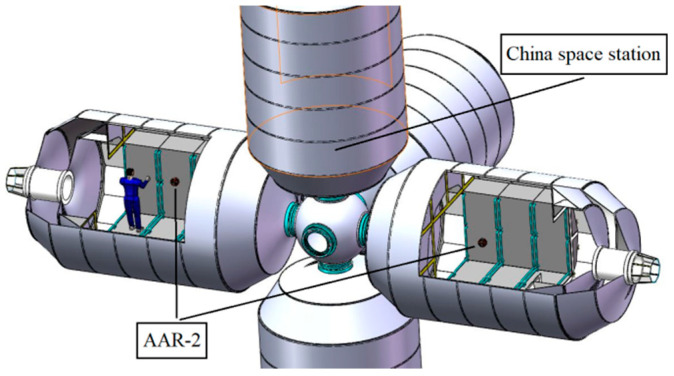
Imagination of AAR-2 running in China Space Station.

On 16 October 2021, the Shenzhou XIII manned spacecraft successfully docked with the Tianhe core module, and then three astronauts entered the Tianhe core module. China’s space station project is in full swing [22]. The astronauts in the space station have very limited time, and the robot in the cabin can provide some help to them. For example, the robot in the cabin can do some repetitive work, such as daily logistics management, searching for needed items, monitoring the air quality, and managing the health of astronauts [23]. Therefore, various free-flying cabin robots have appeared, such as Sphere developed by MIT University; SCAMP made by the University of Maryland; PSA, Smart Sphere [24], and Astrobee [25] developed by NASA AMS Research Center; CIMON made in Germany [26]; Int-ball [27] and Int-ball2 [28] developed by the Japan Aerospace Exploration Agency (JAXA) [29]; and the two generations of astronaut-assistant robots, AAR-1 and AAR-2, developed in Shenyang Institute of Automation, Chinese Academy of Sciences. The imagination of AAR-2 running in China Space Station is shown in Figure 1 [30]. The free-flying cabin robot has no physical contact with the space cabin, and it experiences no mechanical vibration. It operates in a microgravity environment. Moreover, it can operate with minimal power and assist astronauts in conducting scientific experiments [23].

The gaze control is especially suitable for the narrow and busy space station in the microgravity environment. The reasons are as follows. Firstly, the astronauts have very limited time in the space station, and they can free their hands by the gaze interaction. The cabin robots can be controlled by gaze to help astronauts perform repetitive tasks. Secondly, the image acquired by the camera is affected by the distance, and when the camera is far from the robot, the collected image will be relatively small and unclear. The space station is in a narrow region, and the camera installed in it can capture relatively clear robot images in real time, so it is convenient for astronauts to achieve the monitoring and control. Thirdly, in contrast to the ground environment, the microgravity environment in space requires minimal power to make the robot operate. This renders the robot’s operation highly energy-efficient and enables more effective task execution.

### 1.2. Related Work on Gaze Tracking Based on Attention Mechanisms

Gaze tracking technology mimics biological visual processing by employing cameras or sensors to track and analyze gaze trajectories in real-time. Through feature extraction and machine learning models, the system accurately determines gaze targets. This capability enables intuitive user behavior analysis in space HRI, thereby enhancing interaction naturalness. Attention mechanism is a computational approach that enables models to dynamically focus on relevant parts of input data; when applied to gaze tracking, it allows the system to selectively prioritize and process key gaze-related elements, enhancing accuracy and adaptability in gaze tracking. The attention mechanism here refers to the degree of attention rather than the focus of the output gaze. In this mechanism, regions or channels with important information in the image are assigned higher weights, while those with less influence on the output gaze are given smaller weights. There are several common attention mechanisms, such as spatial attention (SA), channel attention (CA), and efficient channel attention (ECA), the structures and principles of which are detailed in [31]. Related work in gaze tracking using attention mechanisms is summarized below.

A gaze tracking approach based on continuous image frames is proposed, in which a bidirectional long short-term memory network and an output attention mechanism are employed to enhance accuracy and real-time performance [31]. Different from the gaze tracking approach based on continuous image frames, in [32,33,34], gaze tracking utilizing still images and attention mechanisms has been investigated. In [32], the face image is used as the input and the spatial attention mechanism is employed to determine a more reasonable gaze point position. The spatial attention mechanism mainly distributes different weights to different regions of the image. A higher weight is given to the region in the face image that has a greater influence on the gaze point position, which can effectively improve the tracking accuracy. In [33], the original complete-image branch is considered as the main branch, and the cascaded branch of face image and face grid is regarded as the weight-contributing branch. The outputs of these two branches are multiplied element-by-element. This operation, which can be seen as an attention mechanism, is used to emphasize the important information that influences the gaze point, thereby improving the accuracy of gaze tracking. This approach is particularly well-suited for gaze tracking in natural scenes, in which the captured image may be of the side face or the back. Due to the lack of detailed eye-related information, which decisively determines the position of gaze points, the accuracy of gaze tracking is not high. In [34], face image, face grid, and binocular images are used as inputs. This compensates for the deficiency of the inputs in the previous two methods. The spatial attention mechanism in [32] and the element-wise multiplicative attention mechanism in [33] are leveraged to take full advantage of their respective strengths. The results validate that the gaze tracking approach based on binocular feature fusion and spatial attention mechanism achieves higher accuracy, as demonstrated in our previous work in [34].

The gaze tracking studied in this paper is not restricted to continuous image frames; it can also take still images as input. Based on the above research, an improved gaze tracking approach is proposed, which provides a theoretical foundation for space HRI based on gaze.

### 1.3. The Main Contribution

In the paper, AAR-2 is used as the experiment platform to realize the simulation of space HRI based on gaze. In the space cabin, the astronaut faces a laptop equipped with a built-in camera, which captures real-time images of the astronaut. Through gaze-based interaction, the astronaut controls the on-screen buttons and operates the AAR-2. The main contributions of this paper are summarized as follows:

(1) Gaze tracking is successfully developed and applied in space HRI. The movement of AAR-2 is controlled by the astronaut’s gaze, and its running state is fed back to the astronaut simultaneously.
(2) Information transmission between the laptop computer and the STM32 microcontroller (a 32-bit controller by ST Microelectronics) in AAR-2 is achieved via radio frequency communication, enabling its free flight.
(3) A gaze tracking database is established, and a highly accurate gaze tracking model based on the attention mechanism is proposed, providing opportunities for space HRI based on gaze.
(4) In the process of image preprocessing, a series of measures are taken to improve the speed, enabling real-time gaze tracking.

The following sections are arranged as follows. In Section 2, the proposed gaze tracking method based on the attention mechanism is introduced. In Section 3, the space HRI experiment is described in detail, which includes the system composition, implementation details (such as database and model establishment, image acquisition, preprocessing and gaze tracking, human–robot interface, radio frequency communication, and ducted fan control), as well as experimental results and analysis. Section 4 presents the conclusions and future work.

## 2. The Proposed Gaze Tracking Approach Based on Attention Mechanism

### 2.1. Proposed Gaze Tracking Method (BinocularFullAttention)

**Figure 2:**
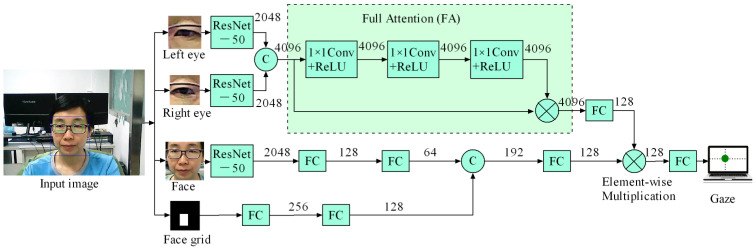
Schematic diagram of proposed gaze tracking method (BinocularFullAttention).

The schematic diagram of the proposed gaze tracking approach denoted as BinocularFullAttention is shown in Figure 2. Here, the channel number is indicated on the arrow, the ‘c’ in the circle stands for cascading operation, and the ‘×’ represents element-wise multiplication.

The system consists of two major branches: the upper one is the binocular branch, and the lower one is the main branch. In the binocular branch, the features of the images from the left eye and the right eye are first extracted by the ResNet-50 network, and then these extracted features are fused by cascading. Next, the proposed full attention (FA) mechanism, which will be described in detail in Section 2.2, is adopted to improve the accuracy. Then, a fully connected (FC) layer is used to reduce the dimension to 128 channels, which serves as the output of binocular branch. In the main branch, the face grid and the feature map, which is extracted from the face image by the ResNet-50, are, respectively, processed by two FC layers. The dimensions of their respective output feature layers are adjusted. After that, they are cascaded together and converted by an FC layer to 128 channels, which serves as the output of the main branch. Finally, the outputs of the main branch and binocular branch are subjected to element-wise multiplication and then transformed by an FC layer to produce the final position of the gaze point on the laptop screen.

### 2.2. Proposed Full Attention Mechanism

The proposed full attention (FA) mechanism is shown in the dotted rectangle of Figure 2. Although FA is somewhat similar in structure to the spatial attention (SA) mechanism in [34], it is quite different in principle. The weight branch is also mainly composed of three cascaded modules, each containing a 1 × 1 convolution followed by a Rectified Linear Unit (ReLU). In the SA mechanism, the output channel number of the weight branch is 1, and the weights of different channels are the same for the same spatial location. In contrast, in the FA mechanism, the output channel number of the weight branch is the same as the input feature map, which is 4096, and the weights of different channels are different for the same spatial location. Through the FA mechanism, different spatial locations and different channels in the same spatial location are all assigned distinct weights, which is equivalent to the integration of spatial attention (SA) and channel attention (CA). The FA mechanism is adopted to suppress the influence of illumination, specular reflection, occlusion, and other interference on gaze tracking accuracy. This enables the model with FA to achieve better accuracy.

### 2.3. Gaze Tracking Comparison Experiments

#### 2.3.1. Comparison Experiments of Gaze Tracking with Other Attention Mechanisms

To verify the effectiveness of the proposed method, the comparison experiments are conducted in the GazeCapture database [35]. The comparison of models with different attention mechanisms on the top 150,000 samples of the GazeCapture database is shown in Table 1.

The first column in Table 1 is the local attention mechanisms adopted, in which N indicates no attention mechanism, CA denotes channel attention mechanism, SA represents spatial attention mechanism, and FA signifies the full attention mechanism. In the second column, Y and N indicate whether the global attention mechanism (GAM) is adopted [34]. The test errors of models with different attention mechanisms are shown in the third column, and the consumed time per epoch is listed in the last column. By comparing the first four rows, it is obvious that the model with the proposed FA mechanism highlighted in bold has the smallest test error and the highest accuracy. By comparing the last four rows, the same conclusion can be drawn. The time consumption for the model with FA is slightly higher than that with SA. As mentioned above, the FA mechanism is equivalent to the integration of the SA and CA mechanisms, so its attention is more comprehensive and effective. Compared with SA, the weight output channels in FA increases from 1 to 4096, and some output parameters are added. However, for the model with SA, the output channel of the weight branch is also increased to 4096 before element-wise product with the major feature. Thus, the computational cost is basically the same as that of the model with FA. For the latter, the processing time of 150,000 images in every epoch is 99.9 min. So the processing time per image can be calculated as follows:

99.9 × 60/150,000 = 0.04.(1)

It is evident that the processing time for each image is 0.04 s. Consequently, there is little difference in processing time between the model with FA and that with SA. However, the improvement in accuracy achieved by the model with FA demonstrates its superiority.

#### 2.3.2. Comparison Experiments with Other State-of-the-Art Gaze Tracking Approaches

Furthermore, the proposed BinocularFullAttention model is compared with the other state-of-the-art methods. Comparison of models on the whole GazeCapture database with 1,490,959 samples is shown in Table 2.

The models in the first two rows are iTracker, proposed by Krafka et al. This model takes a face image, face grid, and left and right eye images as inputs. It does not incorporate any attention mechanisms and achieved a test error of 2.23 before image augmentation; this error was later reduced to 1.93 following the augmentation [35]. The subsequent model is TAT, which primarily adopts an iterative random knowledge distillation approach, resulting in a test error of 1.95 [36]. The following model enhances the iTracker framework by integrating a global attention mechanism (GAM) and incorporating a spatial attention (SA) mechanism within the feature fusion channel for both eyes. This strategic enhancement notably boosts tracking accuracy, resulting in a reduced tracking error of 1.86 [34]. In contrast, the subsequent SpatiotemporalAM model leverages a spatiotemporal attention mechanism but excludes left and right eye images from its inputs, thereby constraining its tracking precision [31]. Finally, the proposed BinocularFullAttention model, much like the one in the fourth row, incorporates a global attention mechanism (GAM). However, a key distinction lies in its substitution of the spatial attention (SA) mechanism with a full attention (FA) mechanism, ultimately resulting in a test error of 1.82 cm. It is evident that the proposed approach achieves the highest accuracy, thereby validating its effectiveness.

## 3. Space Human–Robot Interaction Experiment

The space HRI simulation experiment is performed. The system composition, implementation details, experiment results, and analysis will be demonstrated.

### 3.1. System Composition

**Figure 3:**
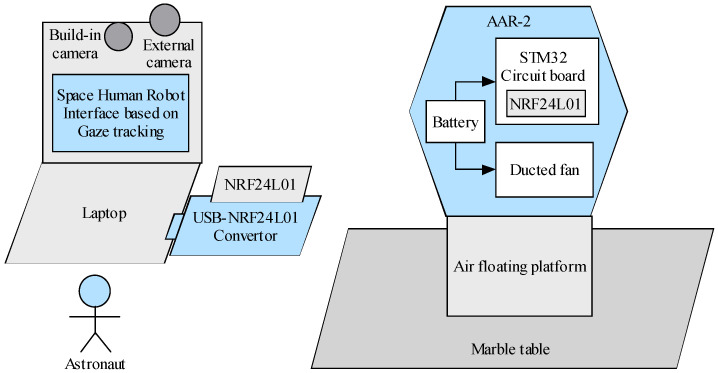
Composition of space human–robot interaction experimental system.

The whole system is mainly composed of astronaut, laptop, camera, nRF24L01 of radio frequency communication module, air floating platform, and AAR-2, as shown in Figure 3. As shown on the right side of the figure, the AAR-2 is mounted on a floating platform which simulates the microgravity environment in space, and AAR-2 can move on the marble table. The AAR-2 is equipped with a circuit board, including STM32 control chip, nRF24L01, and related electronic circuits and devices. The ducted fans are installed inside AAR-2 to drive it. The circuit board and ducted fans are powered by an internal rechargeable lithium battery. As shown on the left side of the figure, the astronaut sits in front of the laptop, and the built-in camera captures the images of the astronaut, while the external camera acquires those of the AAR-2. On the laptop screen, the space human–robot interface based on gaze tracking is displayed, in which the images of the astronaut and AAR-2 are demonstrated in real time. When the astronaut gazes at a button on the interface, it will be automatically clicked to control the AAR-2 to carry out the corresponding action.

### 3.2. Implementation Details

**Figure 4:**
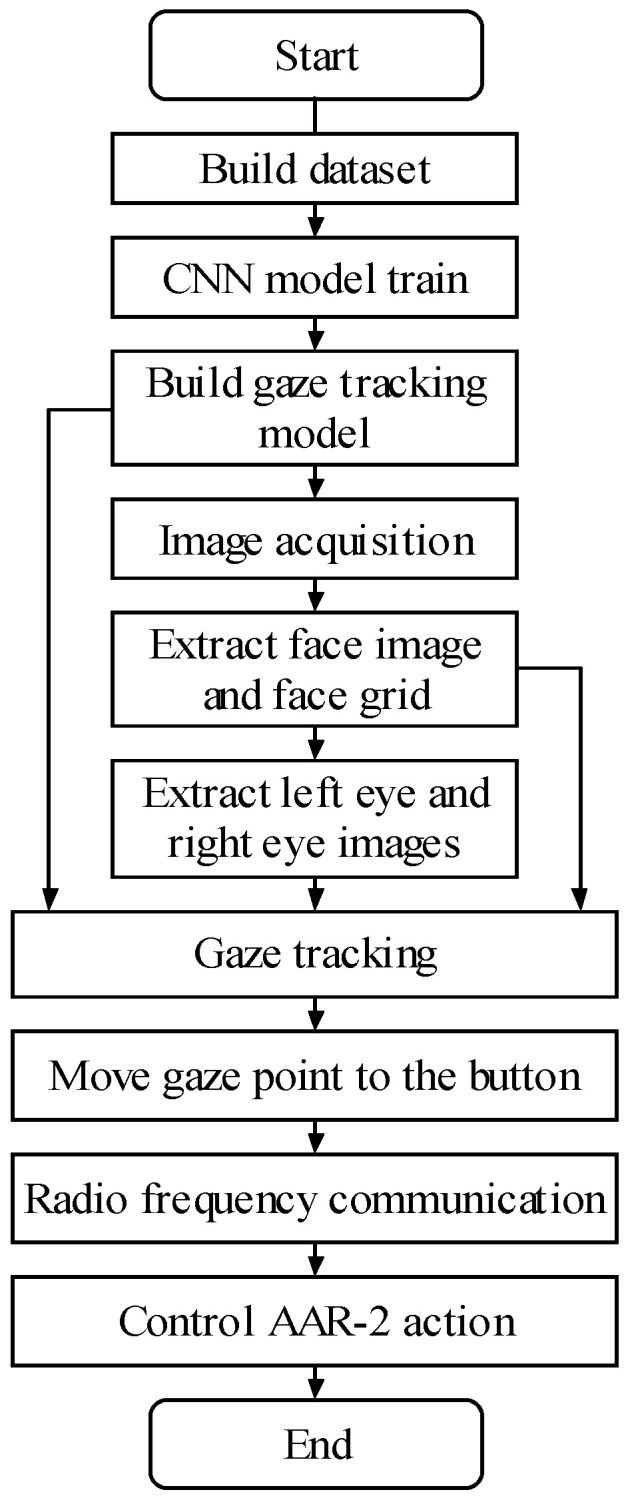
Overall flow chart of human–robot interaction system.

The overall flow chart of the HRI system is shown in Figure 4. Firstly, the gaze tracking database is established, in which the positions of the gaze points on the laptop screen are recorded while the face images are collected. Then the built database is used to train and test the proposed gaze tracking model, and the best one with the least test error is saved. After that, the image is collected in real time, and the face image, face grid, and binocular images are extracted. Then by virtue of the gaze tracking model established in advance, the position of the gaze point is computed, in order to carry out real-time gaze tracking. The space human–robot interface based on gaze tracking is displayed on the screen of laptop. When the gaze point moves to a control button on the interface, the corresponding control instruction is transmitted to the STM32 controller of AAR-2 by radio frequency communication. The controller then sends out PWM signals to adjust the rotation speed of the ducted fans which drive the AAR-2 to act accordingly. The detailed process is described below.

#### 3.2.1. Establishment of Database and Model

**Figure 5:**
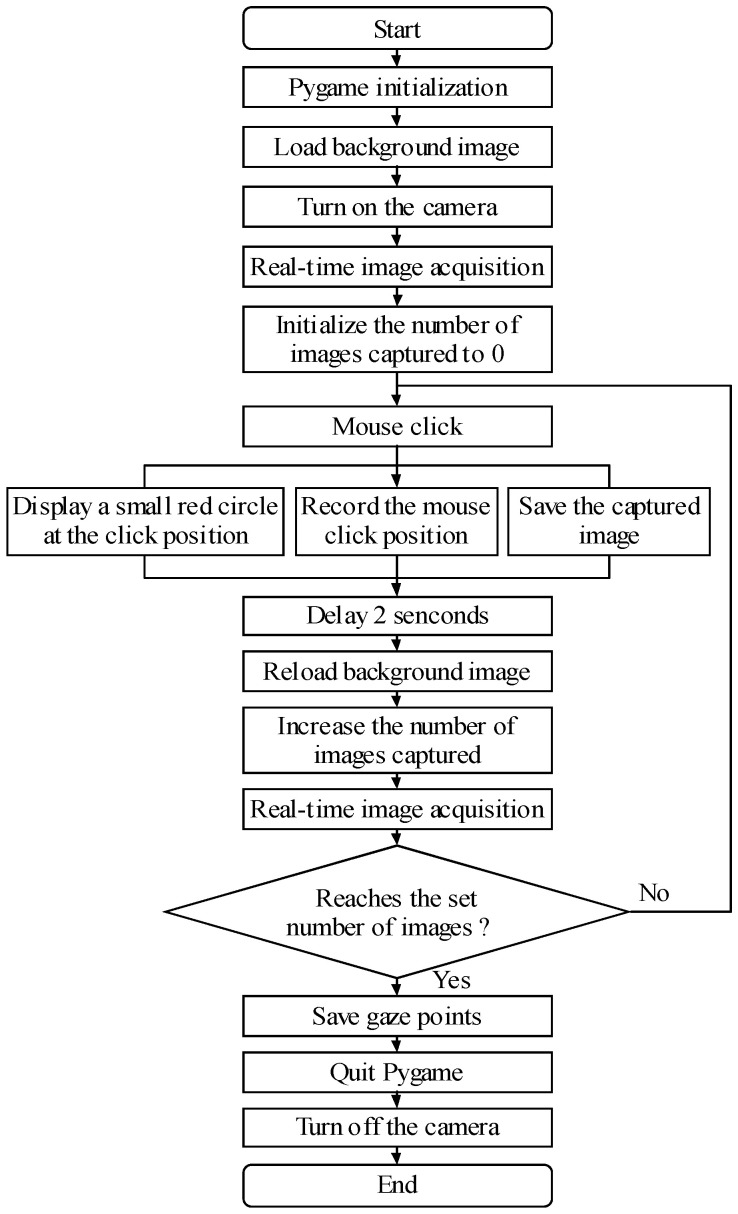
Program flow chart to obtain the images and gaze points.

In the gaze tracking database, there are 20 subjects, and 576 images with the resolution of 640 × 480 are captured per person. A total of 11,520 samples are collected. The program to obtain the database is written in Python 3.5.2, and the program flow chart to obtain the images and gaze points is shown in Figure 5. The background image is designed as a number of small grids. When the mouse clicks a position in the grid, a small red circle will appear at the position where the mouse clicks. When the subject gazes at the small red circle in the mouse click position, the image is captured and saved, and, at the same time, the coordinate of the mouse click position is recorded by virtue of pyGame [37]. After all the samples with images and gaze positions are collected, the gaze tracking database is established. The proposed gaze tracking model, which is based on the FA mechanism, is trained and evaluated on our self-built database to optimize its performance. In this process, 80 percent of the samples in the database, specifically 9216 samples, are randomly selected for training, while the remaining 20 percent, namely 2304 samples, are utilized for testing. To reduce the model’s uncertainty, we adopted a 5-fold cross-validation approach. Specifically, we divided the samples into five subsets, each accounting for 20% of the total samples. During each iteration, we used four subsets (80% of the samples) for training and the remaining one subset (20% of the samples) for validation. The test error, which was used as the evaluation metric, was computed as the mean of the Euclidean distances between the estimated gaze points and the target gaze points. This cross-validation process was repeated five times in total. Subsequently, we computed the average of the validation results from these five iterations to obtain the final test error. Moreover, as the gaze point of the astronaut on the laptop screen is tracked, the pose between the laptop and the astronaut is relatively fixed during the operation. Thus, the built-in camera on the laptop cannot capture the astronaut’s image in an inverted position, but the pose in the direction of pitch, roll, and yaw may be more. In order to solve this problem, in the process of collecting the images, the subjects try to change diverse pose as much as possible, so that the database covers the entire workspace range of practical applications.

#### 3.2.2. Image Acquisition, Preprocessing, and Gaze Tracking

Images are captured by the built-in camera of the laptop for real-time gaze tracking. Then the preprocessing process, that is, extracting face image, face grid, and binocular images, is carried out to prepare the input for the gaze tracking model. There are two methods to extract the face image from the input image, one is to use Dlib library [38], the other is to use OpenCV [39]. For face detection with Dlib library, the accuracy is high, but the speed is low, especially in the Windows system. In contrast, the accuracy of face detection with OpenCV is not as high as that of Dlib, but the speed is higher. For more complex scenes, or side face rather than frontal face, Dlib library is adopted for face detection. While in actual application, the images captured by astronauts sitting in front of a laptop computer are basically frontal face ones, so OpenCV is used for face detection to improve the detection speed. The specific algorithm to determine the face grid region is shown in Algorithm A1 in Appendix A.

In the process of extracting the face image, the position and size of the face image in the original input image are recorded, so as to establish the face grid. The resolution of face grid image is 25 × 25. According to the left top coordinates (Xf, Yf), the width (Wf), and height (Hf) of face ROI (Region of Interest), and the width (Wi) and height (Hi) of the input image, the face grid is calculated. After face image extraction, the left and right eye images are extracted. Although the eye images can also be extracted by OpenCV, its accuracy is too low, so Dlib is adopted to extract them. Firstly, the key landmarks of the human face are obtained by Dlib, and then the eye ROIs are determined by the landmarks around eyes. There are two kinds of face landmarks obtained by Dlib, 68 and 5, respectively. The 68 face landmarks contain 6 landmarks around each eye, while the 5 face landmarks only contain 2 landmarks at two canthus. Although by both kinds of landmarks detection, all left and right eye images can be extracted, in order to improve the real-time performance, the 5 face landmarks detection is selected. The midpoint of the two canthi is taken as the center, 1.5 times the distance between them along the x axis is the width and height of the eye ROI image. The specific algorithm to determine the eye ROI is shown in Algorithm A2 in Appendix B, where its top left coordinate (X, Y) and its width and height (W, H) can be determined by the coordinate of left canthus (Xcl, Ycl) and that of right canthus (Xcr, Ycr). Furthermore, if the extracted eye ROI is beyond the range of the face image, it will be limited. The limiting algorithm of left eye ROI is shown in Algorithm A3 in Appendix C, where its left eye ROI is (Xleft, Yleft), and its width and height are Wleft and Hleft, respectively.

For the ROI of the left eye, when it exceeds the right boundary of the face image, its width is reduced so that the right boundary is that of the face image. At the same time, the eye ROI height is also reduced to keep the width and height equal. When the ROI of the left eye exceeds the upper boundary of the face image, its height is reduced so that its upper boundary is that of the image. At the same time, the eye ROI width is also reduced to maintain the same width and height. In addition, when the ROI of the left eye exceeds both the right boundary and the upper boundary of the face image, the width and height of the image are set as the minimum distance between the left / lower boundary of the ROI and the right/upper boundary of the image, respectively. Similarly, for the right eye ROI, when it exceeds the left boundary of the face image, the left boundary of the right eye ROI is that of the image; when it exceeds the upper boundary, the upper boundary of ROI is that of the image; when it exceeds the upper and left boundary, the width and height of ROI are adjusted to the minimum distance between the right / lower boundary of ROI and the left/upper boundary of image, respectively.

In conclusion, a series of measures have been taken to improve real-time performance in the above preprocessing process. Then the face image, face grid, left eye, and right eye images are fed into the gaze tracking network model to determine the positions of gaze points on the laptop screen, so as to realize real-time gaze tracking.

#### 3.2.3. Human–Robot Interface

**Figure 6:**
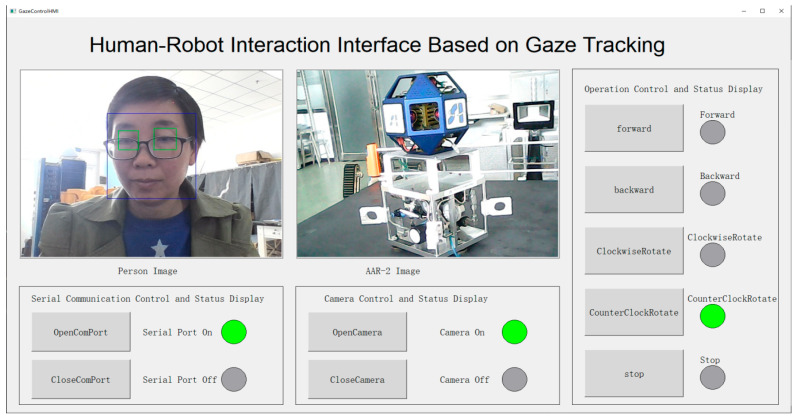
Space human–robot interaction interface based on gaze tracking.

The space human–robot interface based on gaze tracking is shown in Figure 6. The subject image captured by the built-in camera of the laptop is displayed in the left upper corner of the figure. The AAR-2 image captured by the external camera is displayed on the right of the subject image. The face ROI and two eye ROIs are marked by the blue box and green boxes, respectively. Serial port control buttons and status indicator lights are in the left lower corner, and next to them, manual control buttons and status indicator lights of the built-in camera are demonstrated. The rightmost column is the operation control buttons and status indicator lights of the AAR-2. After the program runs, the mouse position is first set to the “OpenComPort” button. At this time, the “OpenComPort” button is automatically clicked, then the communication connection is established, the camera is turned on, images are acquired in real time, and the position of the gaze point is estimated by the gaze tacking model in real time. The mouse position in the monitor varies along with the actual gaze point. When the gaze is located on an operation control button on the right side for 2 s, the button will be automatically clicked and the corresponding control instruction will be transmitted to the STM32 controller in AAR-2 by radio frequency communication, so as to realize the corresponding control. At the same time, the status of AAR-2 is fed back to the laptop and displayed by the indicator light on the right side of the button. The forward and backward movement, counterclockwise and clockwise rotation, stop, and the corresponding status display of AAR-2 are realized. As shown in Figure 6, this is the human-robot interaction simulation interface when controlling the counterclockwise rotation of AAR-2 through gaze. Please refer to the Supplementary Materials for the corresponding video.

#### 3.2.4. Radio Frequency Communication

**Figure 7:**
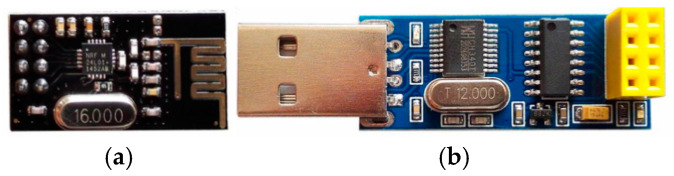
Communication module: (**a**) nRF24L01 radio frequency module; (**b**) USB-nRF24L01 conversion module.

In the whole system, the laptop serves as the upper computer, while the STM32 controller in AAR-2 acts as the lower computer. On the upper computer, the gaze tracking network model, and HRI interface are developed using Python. As for the lower computer, programs for controlling the movement of AAR-2 are developed in the Keil uVision5 development environment with the C language. The upper and lower computers communicate by the nRF24L01 radio frequency module, as shown in Figure 7a. In the upper computer, because there is not a nRF24L01 radio frequency module interface but a USB serial port, the USB serial port signals need to be converted into radio frequency signals. To this end, USB-nRF24L01 communication conversion module, shown in Figure 7b, is adopted to convert the signals. Then the signals are transmitted to the lower computer by the radio frequency module. After that, the lower computer sends the corresponding instructions to control the operation of the ducted fans, so as to drive the AAR-2 to carry out the corresponding action.

In order to realize the wireless communication between the upper and lower computers, it is necessary to keep the communication-related parameters consistent. In the upper computer, the serial port debugging assistant software, XCOM V2.0 is first utilized to set the relevant parameters of the USB wireless serial port. The baud rate is set to 115,200, the module transmission rate is set to 2 Mbps, the local receiving address and the target board address are both set to 0x34, 0x43, 0x10, 0x10, and 0x01, the communication frequency is set to 2.440 GHz, and the CRC check code is set to 16 bits. Then in the Python program of the upper computer, the relevant communication parameters are set. The above communication parameters in the lower computer correspond to those in the upper computer. When the upper computer sends seven bytes of hexadecimal data, the lower computer will receive eight bytes of ASCII code, in which the first byte is the bytes number of the data, and the remaining seven bytes are the data. On the contrary, when the lower computer feedbacks the running state to the upper computer, eight bytes of ASCII code are sent, in which the first byte is the bytes number of data sent, while seven bytes of data are received in the upper computer.

The communication protocol is defined as follows. The control instruction sent from the upper computer to the lower computer is shown in Table 3. The status information sent from the lower computer to the upper computer is shown in Table 4.

The formats of the control word and the status word are similar, except that the first byte in the lower computer is the bytes number (BN) of data, as shown in Table 4 (status information). The start byte is set to aa in hexadecimal or 170 in ASCII. Then it is the control or status flag byte, f9 in hexadecimal is the control flag, and 9 in ASCII is the status flag. The middle is four bytes of data, which is the control command sent from the upper computer to the lower computer, or the running state fed back from the lower computer to the upper computer. The ff in hexadecimal or 255 in ASCII in the middle four bytes successively represents forward movement, backward movement, counterclockwise rotation, and clockwise rotation. When the values in the middle four bytes are all 0, it means stop. The last end byte is set to dd in hexadecimal, or 221 in ASCII.

#### 3.2.5. Control and Drive of Ducted Fan

**Figure 8:**
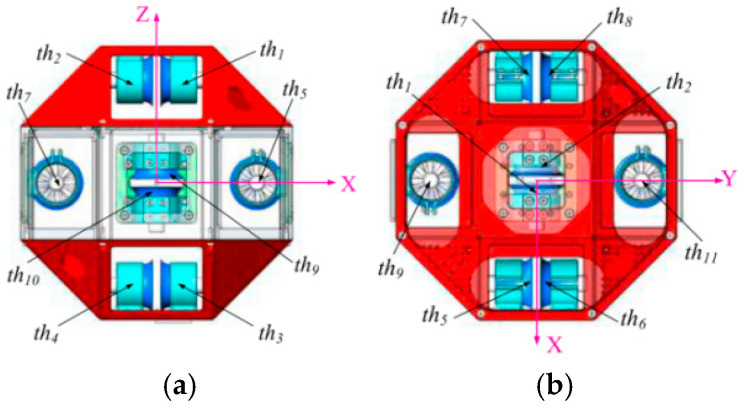
The views of AAR-2: (**a**) Left view; (**b**) top view.

The left view and top view of AAR-2 are shown in Figure 8 [30].

From Figure 8a of the left view of AAR-2, it can be seen that when the rotation speeds of 2 and 4 ducted fans are increased, AAR-2 moves forward, and when those of 1 and 3 ducted fans are increased, the AAR-2 moves backward. From Figure 8b of the top view of AAR-2, it can be seen that when the speeds of 5 and 8 ducted fans are increased, AAR-2 rotates counterclockwise, while when the speeds of 6 and 7 ducted fans are increased, AAR-2 rotates clockwise. Furthermore, since AAR-2 flies freely in the space station, it can carry out three degrees of freedom (DOF) translation and three DOF rotation. In the experiment on the ground, only one DOF translation and one DOF rotation are simulated. In the actual space station, in order to realize translation and rotation in other directions, the robot just needs to adjust the rotation speed of the corresponding ducted fans. In order to achieve left or right translation of AAR-2, it only needs to simultaneously control the speed of fans 5 and 7 or fans 6 and 8. If AAR-2 shifts up or down, it only needs to regulate the speed of fans 10 and 12 or fans 9 and 11. Similarly, rolling and pitching operations can also be realized.

### 3.3. Experimental Results and Analysis

#### 3.3.1. Comparative Analysis of Gaze Tracking Models with Various Attention Mechanisms

Experiments are conducted on the above self-built database for models with different attention mechanisms, and the comparison results are shown in Table 5. From the first four rows or the last four ones, it can be seen that the models with FA are better than those with CA or SA. As can be seen from the last row, the proposed approach has the highest gaze tracking accuracy, which has the same conclusion as on the GazeCapture database. The time cost of 11,520 images in every epoch is 7.9 min, so the time taken for each image can be computed as follows:

7.9 × 60/11,520 = 0.04 s.(2)

For each image, the time cost of the model employing FA is 0.04 s, which is nearly identical to that of the model utilizing SA.

To sum up, based on the analysis of experimental results on a self-made database, the accuracy of the proposed gaze tracking approach outperforms others and meets the requirements of real-time performance, which once again validated the effectiveness of the proposed FA mechanism. Moreover, the generalization ability of the proposed BinocularFullAttention model is also verified.

**Table 5:** Comparison of models with different attention mechanisms on self-constructed database.

Local Attention Mechanism	Global Attention Mechanism	Test Error (cm)	Time (Minute) /Epoch
N	N	0.3912	6.0
CA	N	0.3638	6.6
SA	N	0.3248	7.6
FA	N	0.3022	7.9
N	Y	0.3737	6.0
CA	Y	0.3552	6.8
SA	Y	0.3090	7.8
FA	Y	0.2852	7.9

#### 3.3.2. Physical Scene Visualization in Space Human–Robot Interaction Simulation Experiment

**Figure 9:**
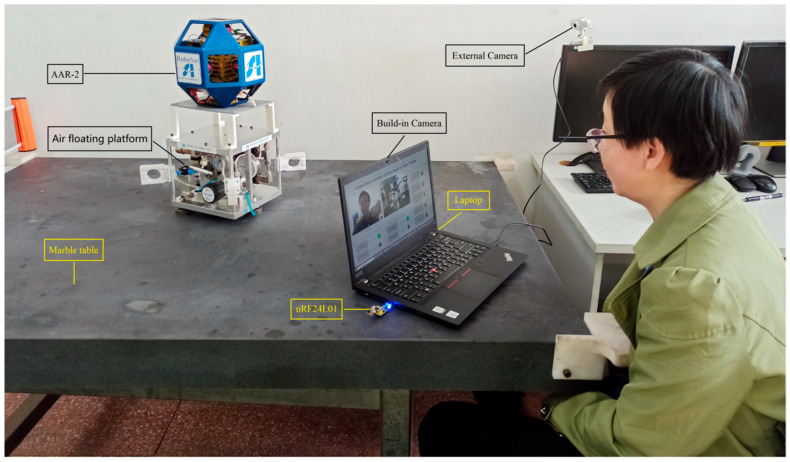
Physical image of space human–robot interaction simulation experiment.

Physical image of space HRI simulation experiment is shown in Figure 9. AAR-2 is mounted on the air floating platform, which can simulate the microgravity environment in space. It can move on the marble table under the drive of a small force. The subject sits in front of the laptop. The space human–robot interface based on gaze tracking is displayed on the laptop screen, in which AAR-2 can be monitored. The subject’s images are captured in real-time via the built-in camera of the laptop, and the gaze point position is calculated in real-time using the internal gaze tracking model. When the subject gazes at different control buttons on the interface, by virtue of nRF24L01 of radio frequency communication, AAR-2 can be controlled to perform different actions. It mainly realizes forward and backward movement, counterclockwise and clockwise rotation, and stop control. At the same time, the running status of AAR-2 is displayed on the screen. For example, when the subject stares at the “backward” button for 2 s, the AAR-2 automatically moves back, and the “Backward” indicator light turns green. When the subject stares at the “stop” button for 2 s, the AAR-2 stops running and the “Stop” light turns red. The control button is designed to be 4 cm wide and 2 cm high. The test error of the adopted gaze tracking model is 0.3 cm. It is clear that when the gaze point moves to most regions on the control button, the actual position of the gaze point will be on the control button, so as to send corresponding control instructions. In the actual operation, the subject usually moves the gaze point to the middle position of the control button, and the above test error is enough to meet the actual accuracy requirements. Moreover, with the movement of the subject’s gaze, the gaze point on the interface can follow the movement in the form of an arrow, and the real-time performance can also meet the actual needs. Furthermore, the staring time can be set and modified according to actual needs.

In summary, a preliminary exploration of gaze-based space HRI was carried out in this paper, and its simulation experiment was conducted. The microgravity environment in space allows a tiny power to drive AAR-2, so compared with the ground environment, space HRI system is more energy-saving, and has better real-time performance.

### 3.4. Discussion

To sum up, the gaze-based space HRI simulation experiment has been implemented. The main goal is to send control commands by the monitoring screen to achieve remote operation of the AAR-2. Therefore, only the 2D gaze points of astronauts on the computer screen need to be tracked, without needing to conduct 3D gaze tracking. Due to the microgravity environment of space, AAR-2 can be propelled with only a little power, making space HRI more energy-saving and more efficient than on Earth. However, the simulation experiments are somewhat rough. In the future, more in-depth research on the gaze tracking will continue to be conducted in order to develop more accurate and real-time approaches and apply them in the space HRI. At the same time, the grippers will be installed on the AAR-2 to assist astronauts in performing tasks within the operational scope of the grippers, thereby better facilitating space human–robot interaction.

## 4. Conclusions

In space, astronauts’ time is extremely valuable, and cabin robots can help astronauts do some repetitive work, monitor their health and so on. Free-flying space robots have unique advantages in the space station because they can operate in a microgravity environment using minimal power. In this paper, AAR-2, a free-flying astronaut-assistant robot in the cabin, is used as the experimental platform. The built-in camera on the laptop is used to collect the image of the astronaut in real time, and the gaze of the astronaut is tracked in real time to achieve space HRI. We establish the gaze tracking database to provide data support for modeling and develop the space human–robot interface based on gaze tracking. When the astronaut gazes at the control button in the interface, the control instruction is transmitted to AAR-2 via radio-frequency communication. Driven by the ducted fans, AAR-2 performs the corresponding action, mainly realizing forward and backward movement, counterclockwise and clockwise rotation, and stop operation. We employ the proposed BinocularFullAttention model, a gaze tracking model based on a full attention mechanism, and the test error is 0.3 cm. The image preprocessing method with high real-time performance is adopted, and the position of the gaze point on the screen can move with the subject’s gaze in real time. The experimental results show that the accuracy and real-time performance of gaze tracking can meet the actual demand.

## Figures and Tables

**Table 1:** Comparison of models with different attention mechanisms on the 150,000 samples of GazeCapture database.

Local Attention Mechanism	Global Attention Mechanism	Test Error (cm)	Time (Minute) /Epoch
N	N	2.4255	78.1
CA	N	2.3920	86.0
SA	N	2.3831	98.9
**FA**	**N**	**2.3623**	**99.3**
N	Y	2.3839	78.4
CA	Y	2.3522	88.6
SA	Y	2.3306	99.7
**FA**	**Y**	**2.3112**	**99.9**

**Table 2:** Comparison of models on the whole GazeCapture database.

Model	Test Error (cm)
iTracker without augment [35]	2.23
iTracker with augment [35]	1.93
TAT [36]	1.95
SA + GAM [34]	1.86
SpatiotemporalAM [31]	1.92
BinocularFullAttention	1.82

**Table 3:** Control instruction.

Start Byte	Control Flag	Forward Move	Backward Move	Counterclockwise Rotate	Clockwise Rotate	End Byte
aa	f9	ff/00	ff/00	ff/00	ff/00	dd

**Table 4:** Status information.

BN	Start Byte	Control Flag	Forward Move	Backward Move	Counterclockwise Rotate	Clockwise Rotate	End Byte
7	170	9	255/0	255/0	255/0	255/0	221

## Data Availability

The data that supports the findings of this study are available in this article. All other public datasets used are available and cited in the references.

## Supplementary Materials

The following supporting information can be downloaded at: https://www.mdpi.com/article/10.3390/biomimetics11020103/s1, Video S1: Video of Simulation Experiment on Gaze-Based Space Human-Robot Interaction: Rotation Control.

## Abbreviations

The following abbreviations are used in this manuscript:

AAR-2	The Second-Generation Astronaut-Assistant Robot
BRI	Brain–robot interface
CA	Channel attention
DOF	Degrees of Freedom
ECA	Efficient channel attention
EEG	Electroencephalogram
FA	Full attention
FC	Fully connected
GAM	Global attention mechanism
HRI	Human–robot interaction
ReLU	Rectified Linear Unit
ROI	Region of Interest
SA	Spatial attention

## Appendix A

**Algorithm A1**: Determin face grid region	
Input:	
	The width and height of Input image: Wi, Hi The left top coordinate of face ROI: Xf, Yf The width and height of face ROI: Wf, Hf

Compute process:	
	factor 1 = 25/Wi factor 2 = 25/Hi XGrid = Xf ∗ factor 1 YGrid = Yf ∗ factor 2 WGrid = Wf ∗ factor 1 HGrid = Hf ∗ factor 2

Output:	
	The left top coordinate of face grid: XGrid, YGrid The width and height of face grid: WGrid, HGrid

## Appendix B

**Algorithm A2**: Determin eye ROI	
Input:	
	The coordinate of left canthus: Xcl, Ycl The coordinate of right canthus: Xcr, Ycr

Compute process:	
	width = Xcr − Xcl midY = (Ycl + Ycr)/2 X = Xcl − 0.25 ∗ width Y = midY − 0.75 ∗ width W = 1.5 ∗ width H = W

Output:	
	The left top coordinate of eye ROI: X, Y The width and height of eye ROI: W, H

## Appendix C

**Algorithm A3**: Left eye ROI limitation	
Input:	
	The width of face image: Wf The left top coordinate of left eye ROI: Xleft, Yleft The width and height of left eye: Wleft, Hleft
if(Xleft > Wf − Wleft and Yleft >= 0) then{ Wleft = Wf − Xleft Hleft = Wleft } if(Yleft < 0 And Xleft <= Wf − Wleft) then{ Hleft = Hleft + Yleft Wleft = Hleft Yleft = 0 } if(Yleft < 0 And Xleft > Wf − Wleft) then{ if(Yleft + Hleft < Wf − Xleft) then{ Hleft = Hleft + Yleft Wleft = Hleft Yleft = 0 } else { Wleft = Wf − Xleft Hleft = Wleft Yleft = 0 } }	
Output:	
	The left top coordinate of left eye ROI: Xleft, Yleft The width and height of left eye ROI: Wleft, Hleft
